# A critical review of wastewater-based epidemiology as a tool to evaluate the unintentional human exposure to potentially harmful chemicals

**DOI:** 10.1007/s00216-024-05596-z

**Published:** 2024-10-18

**Authors:** Rodrigo B. Carneiro, Maria-Christina Nika, Rubén Gil-Solsona, Konstantina S. Diamanti, Nikolaos S. Thomaidis, Lluís Corominas, Pablo Gago-Ferrero

**Affiliations:** 1 Laboratory of Chromatography, São Carlos Institute of Chemistry (IQSC), University of São Paulo (USP), 400, Trabalhador São-Carlense Ave., São Carlos, São Paulo 13566-590 Brazil; 2 Department of Environmental Chemistry, Institute of Environmental Assessment and Water Research (IDAEA), Severo Ochoa Excellence Center, Spanish Council of Scientific Research (CSIC), Jordi Girona 18-26, E-08034 Barcelona, Spain; 3 Laboratory of Analytical Chemistry, Department of Chemistry, National and Kapodistrian University of Athens, Panepistimiopolis Zografou, 15771 Athens, Greece; 4 Catalan Institute for Water Research (ICRA-CERCA), Emili Grahit 101, 17003 Girona, Catalonia Spain; 5 University of Girona, Plaça de Sant Domènec 3, 17004 Girona, Catalonia Spain

**Keywords:** Mass spectrometry, Wastewater analysis, Population normalized mass load, Human biomonitoring, Biomarkers of exposure, Human health

## Abstract

Wastewater-based epidemiology (WBE) is a powerful tool to gather epidemiological insights at the community level, providing objective data on population exposure to harmful substances. A considerable portion of the human exposure to these potentially harmful chemicals occurs unintentionally, unlike substances such as pharmaceuticals, illicit drugs, or alcohol. In this context, this comprehensive review analyzes WBE studies focused on classes of organic chemicals to which humans are unintentionally exposed, namely organophosphorus flame retardants, per- and polyfluoroalkyl substances (PFAS), benzotriazoles and benzothiazoles, phthalates and terephthalates, benzophenones, pesticides, bisphenols, and parabens. The review highlights some advantages of WBE for public health surveillance, e.g., non-invasive analysis, predictive capability, nearly real-time data, population-wide insights, no ethical approval, and unbiased sampling. It also discusses challenges and future research directions in WBE regarding exposure to harmful chemicals from various sources. The review emphasizes the critical role of wastewater sampling, sample preparation, quality control, and instrumental analysis in achieving accurate and reliable results. Furthermore, it examines the selection of human biomarkers for WBE studies and explores strategies to link WBE with human biomonitoring (HBM), which together enhance both the precision and effectiveness of exposure assessments.

**Supplementary Information:**

The online version contains supplementary material available at 10.1007/s00216-024-05596-z.

## Introduction

Every day, population faces increased exposure to a myriad of chemicals that bear high relevance to human health and may pose a significant threat to the development of diseases. Over the years, there has been a noticeable rise in conditions like infertility, allergies, neurological disorders, and even various types of cancers. There are several factors that can influence the worsening of these pathologies, but chronic exposure to harmful chemicals has been reported as a high-risk factor for their development [1–5].

Human exposure to harmful chemicals is usually assessed by human biomonitoring (HBM), which involves the analysis of a given set of biomarkers in specimens collected from individuals, such as blood, urine, and hair, among others. However, HBM frequently faces a variety of challenges, including high costs, selection bias (difficulties in selecting individuals representative of an entire population), lack of engagement of cohort participants, ethical approval requirements, and lack of temporal dimension (individuals are usually sampled only once or, at best, over a 24-h period) [6, 7]. Therefore, there is an urgent need to develop alternative approaches to complement HBM studies, for instance, by assessing human exposure to harmful chemicals at community (population) level.

Wastewater-based epidemiology (WBE) is a conceptually simple approach to obtain epidemiological information at the community level by measuring an array of chemical and biological markers for exposure and health status in wastewater samples [8]. This approach can provide objective information on the harmful chemicals to which a population is exposed. It relies on the principle that human biomarkers, i.e., the metabolites generated from organic compounds absorbed by human body, are excreted in urine by individuals into urban sewer networks [9, 10]. WBE has already been widely used to study lifestyle choices and estimate the consumption of illicit drugs, alcohol, prescription, and over-the-counter pharmaceuticals [10–13]. The most recent application of WBE is the worldwide surveillance of the SARS-CoV-2 virus in wastewater for monitoring the prevalence of COVID-19 in the population connected to sewer networks [8, 14–16].

WBE studies might offer various key advantages for public health surveillance, including the following: (i) Non-invasive chemical analysis: WBE serves as a non-invasive method for monitoring public health by analyzing chemical markers present in wastewater; (ii) Predictive capability: WBE facilitates the early detection of spatial and temporal trends in public health, providing valuable insights into existing patterns and potential outbreaks; (iii) Nearly real-time data: By employing biosensors, WBE can provide data in near-real time, enabling swift responses to emerging health threats; (iv) Population-wide insights: WBE offers information on entire populations connected to sewer systems, providing a comprehensive view of public health status; (v) Ethical approval not typically required: Unlike traditional medical studies, WBE studies do not require ethical approvals, if performed at wastewater treatment plant level, streamlining the research process; (vi) Unbiased sampling: Wastewater inherently reflects the collective behaviors and health status of a population; (vii) Comprehensive analysis: WBE enables simultaneous analysis of a wide range of parameters and health factors, offering a holistic view of public health; or (viii) Correlation discovery: Wastewater analysis allows for the identification of correlations between different pharmaceuticals and drugs, offering valuable insights into drug usage patterns and public health trends [7, 17].

**Fig. 1:**
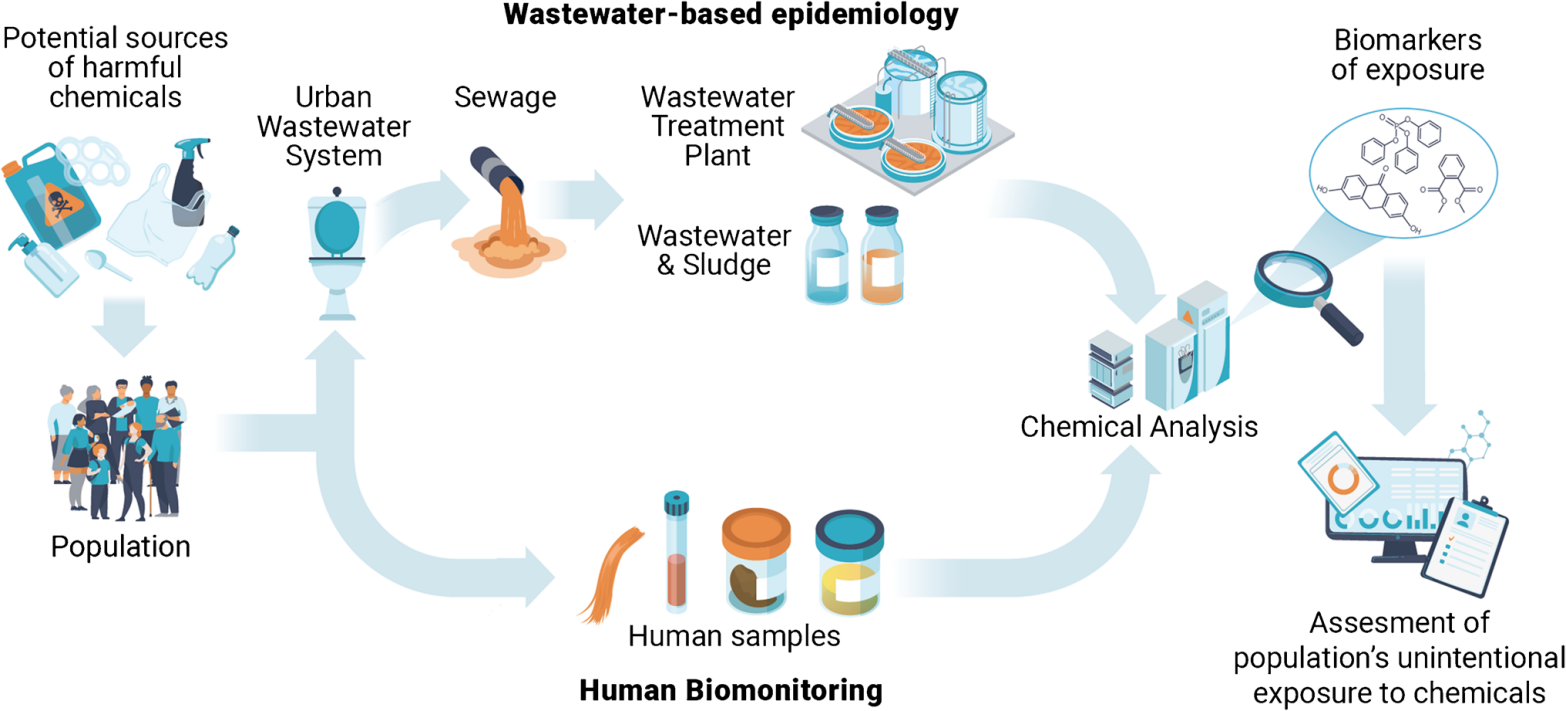
Conceptual framework of wastewater-based epidemiology related to the unintentional human exposure to harmful chemicals and its link with human biomonitoring

However, few studies [6, 18–21] have been undertaken to measure human biomarkers of exposure to harmful chemicals in sewage. In addition, the full potential of WBE to assess exposure to chemicals through air, food, water, and various consumer products (e.g., personal care products, cleaning products, cookware, clothing, and paints) remains underexplored. Notably, the exposure to harmful chemicals (e.g., PFAS or parabens) from these sources is unintentional, unlike other direct consumer products such as pharmaceuticals, alcohol, tobacco, and illicit drugs. Figure 1 schematically illustrates the concept of WBE concerning the unintentional human exposure to harmful chemicals and its link with HBM.

## Scope of the literature review

Articles addressing the application of WBE to chemical exposure primarily focus on intentional human exposure, such as pharmaceuticals [9, 10, 21], illicit drugs [13, 22], or alcohol [11, 23], which are positively correlated with population size and generally intrinsic to human metabolism. Recently, Kasprzyk-Hordern et al. [24] discussed the rationale for integrating WBE and HBM to attain a more comprehensive understanding of chemical exposure. The present article expanded upon this discussion by critically reviewing the application of WBE related to unintentional human exposure to harmful chemicals. Our focus encompassed the analytes under study, methodologies for representative sampling at the community level (including neighborhood-level resolution), sample preparation protocols for efficient extraction of biomarkers, quality assurance/quality control (QA/QC) procedures for extracting reliable results, analytical techniques for both targeted and non-targeted analysis, and other pertinent aspects essential for the optimal exploitation of this technology’s considerable potential.

The selection of proper biomarkers that are specific to human metabolism (or have minimal exogenous sources) in WBE studies represents a key challenge for the validation of this epidemiological tool. The main requirements for a WBE biomarker are the following: being excreted in consistent amounts (preferably in urine) at a rate proportional to exposure, being detectable in urban wastewater, being stable in sewage (both during the transport in sewer and during sampling, storage, and analysis), and having human excretion as a unique source [20, 25]. Considering the unintentional exposure to chemicals that are harmful to human health, this challenge is even greater, since there are multiple sources that contribute contamination to the sewage flowing into the wastewater treatment plants (WWTPs), for example, pesticides and industrial chemicals, among others. Furthermore, there is a lack of understanding of human metabolism for compounds that are not intended for human consumption, making the proposition of endogenously formed markers of exposure to harmful chemicals unfeasible [26].

This review focuses on eight classes of harmful chemicals to which humans are unintentionally exposed, each with documented adverse effects on human health, as listed in Table 1. These include organophosphorus flame retardants, per- and polyfluoroalkyl substances, benzotriazoles and benzothiazoles, phthalates and terephthalates, benzophenones, pesticides, bisphenols, and parabens. Based on these classes of compounds, we searched the “Web of Science” database to select papers addressing factual WBE studies, including pollutant concentration values in sewage and mass load levels (directly or indirectly, in the main manuscript or in supporting information files). The routes of entry into the human body for these harmful chemicals vary from one class to another, but generally refer to the inhalation of contaminated air or dust, dermal contact, mainly for personal care products, and ingestion of contaminated food/water or occasional ingestion of dust particles on surfaces.

**Table 1:** Sources of unintentional exposure to harmful chemicals and their potential effects on human health

Class	Applications and sources of human exposure	Main reported human health effects	References
Phthalates	Manufacturing and processing of plastics, production of polyvinyl chloride products, personal care products (cosmetics, fragrances, perfumes, lotions, shampoos, and nail polish), medical devices (tubing, gloves), toys and childcare products, food packages	Children obesity, lower semen quality, neurodevelopmental issues, increased risk of childhood asthma, allergic symptoms and hypertension	[27–29]
Pesticides	Agriculture, forestry and landscaping to control weeds and pests; residential use to control insects, rodents, and weeds; public health to control vectors of diseases (e.g., malaria)	Chronic neurotoxicity, infant methemoglobinemia, various cancers, immunologic abnormalities, and reproductive and developmental issues	[30, 31]
Flame retardants	Electronic devices (e.g., computers, TVs, and smartphones); furniture and upholstery; textiles (e.g., curtains, carpets, and clothing); building materials (e.g., insulation, foam boards, and coatings); automotive interiors; children’s products (e.g., car seats, strollers, and high chairs)	Endocrine and thyroid disruption, immune system impacts, reproductive toxicity, reproductive health issues, immunological, oncological, and cardiovascular diseases	[32, 33]
Bisphenols	Water bottles, food containers, eyeglass lenses, medical devices, epoxy resins (used in coatings, adhesives, can linings, dental sealants), thermal paper used for receipts, tickets, and labels; electronic components and casings, dental sealants and composites, consumer electronics, automotive parts	Breast cancer, diabetes, reproductive and developmental issues, metabolic disease, and obesity in children	[29, 34, 35]
Per- and polyfluoroalkyl substances (PFAS)	Water and stain repellents, non-stick cookware, food packaging, firefighting foam, electronics and aerospace industry, cleaning products, medical devices	Infertility, hormone disruption, thyroid, liver, and kidney disorders, metabolic dysfunction, and cancer	[36, 37]
Benzotriazoles and benzothiazoles	Corrosion inhibitors, UV stabilizers, antifade agents, lubricants and fuels, photographic chemicals, plastic and rubber products, dyes and pigments	Respiratory irritation, dermal sensitization, mutagenic, neurotoxic and carcinogenic potential	[38, 39]
Benzophenones	UV absorbers in sunscreens, plastic and polymer, stabilizers, fragrances and flavorings, coatings and inks, electronics	Contact and photocontact allergy reactions, endocrine disruption, potential association with cancer and diabetes	[40, 41]
Parabens	Personal care products, cosmetics, pharmaceuticals, food products, industrial products	Male infertility, adipogenesis, hypersensitivity reactions, genotoxicity and carcinogenesis	[42–44]

## Outcomes from the review on the different steps from sampling to analysis

### Sampling strategies and techniques

In WBE studies, the sampling method is crucial, as it has the potential to introduce significant uncertainties into the results. Ort et al. [45, 46] and Aymerich et al. [47] demonstrated that depending on the sampling approach (e.g., grab, flow-based composite, volume-proportional), concentrations of target chemicals in wastewater can vary by orders of magnitude, thus highly affecting the community’s exposure calculations. This variability is particularly pertinent for compounds present at low concentrations and excreted by a small portion of a population, characterized as having low “pulses” according to the nomenclature employed by Ort et al. [45, 46]. The majority of WBE studies reviewed herein, focusing on biomarkers of exposure, utilized 24-h composite samples. However, the details of these composite samples — whether they were based on flow, volume, or time — are often not clearly defined in the literature. Only one study opted for grab sampling methods [48], which are prone to introducing significant errors in the resultant concentration measurements. The other WBE studies (detailed in Supporting Information, Tables S1–S8) were conducted by sampling at the entrance of WWTPs, where it is easy to install autosamplers and accurate flowmeters are usually available. Such setup reduces uncertainties when calculating PNMLs (population normalized mass load, detailed in the “Population normalized mass load related to harmful chemicals” section).

A few studies have ventured into intra-urban WBE by sampling within a city, often at sewer holes. These studies aim to capture intra-city variability by sampling closer to households upstream in the sewer system, which allows for the assessment of different subpopulations. This method accounts for diverse community behaviors and varying levels of exposure to contaminants. Notably, from the review, only one study provided results of unintentional exposure to chemicals such as plasticizers, parabens, and UV filters after sampling in homogeneous urban areas. This study relates to the SCOREwater project (scorewater.eu), which employed strategically positioned sampling stations [49]. Preliminary findings from this project suggest variations in unintentional exposure to chemicals across different neighborhoods, as evidenced by partial results published at Senta et al. [50] and a summary available through https://scorewater.institutmetropoli.cat.

An important aspect to consider is the duration and frequency of sampling campaigns. For biomarkers with variability across seasons, weekdays, and weeks, sampling over an entire year at 13-day intervals is recommended. For compounds with low variability, a 1-week average can represent the annual mean. However, determining the ideal sampling frequency upfront remains challenging, as studies collecting frequent samples over a year are necessary to identify the optimal approach. This is still an unresolved issue.

### Sample preparation, quality control and instrumental analysis

#### Sample extraction and analytes preconcentration

Solid-phase extraction (SPE) was the preferred technique for the preconcentration and purification of wastewater in the reviewed studies for all investigated families of chemicals. One critical aspect during the SPE is the selection of sorbents, as it can significantly affect analyte recovery and method sensitivity. Polymeric sorbents, such as HLB, were the predominant choice in the reviewed studies. Their versatility allowed for the extraction of various classes of target analytes, including bisphenols [51, 52], phthalates [53, 54], parabens [55], and pesticides [25]. C18 sorbent was exclusively utilized by Been et al. [56, 57] for the determination of flame retardants, whereas ion exchange sorbents were selected in only a few studies [58–60], with weak anion exchange sorbents notably proven as the most efficient choice for extracting PFAS [61, 62]. In one study [63] employing a wide-scope screening method for multiple classes of chemicals, a generic extraction protocol was followed. To ensure efficient recovery of compounds with diverse physicochemical properties, in-house cartridges containing four different sorbents, including polymeric and ion exchange materials, were prepared [63, 64]. However, the preparation of cartridges with multi-layer sorbents in the laboratory entails additional costs, is time-consuming, and increases the risk of contamination of any compound of interest. Furthermore, two studies examining a population’s exposure to PFAS employed two cartridges sequentially for further clean-up of the extract [61, 62]. Moreover, in a recent study by Senta et al. [65], the applicability of a simple and fully automated on-line SPE LC–MS/MS approach was investigated in a WBE context, for the determination of 27 biomarkers from different classes of personal care and household products, as an alternative to off-line SPE. The methodology notably reduced analysis time, enhanced the robustness and repeatability of the process, and yielded reliable results. However, the study acknowledged that commonly used targeted off-line SPE methods, which are specifically optimized and validated for single classes of contaminants, demonstrated superior sensitivity, in terms of lower limits of detection (LOD). However, in preliminary experiments (results not shown in the cited paper), commonly used sorbents like HLB, WAX, MAX, and MCX exhibited poor recovery for selected highly polar alkyl phosphate pesticides, and thus, Rousis et al. [25] resorted to direct injection of the samples without any pre-treatment for their determination.

The preconcentration factor employed in extraction protocols plays a crucial role, particularly in heavy matrices such as wastewater, as it significantly impacts overall method performance, particularly in terms of sensitivity and accuracy. While a high preconcentration factor may result in lower limits of detection (LODs), it can also lead to significant matrix effects and signal suppression, ultimately compromising analyte sensitivity and resulting in higher LODs. Among the reviewed studies, the initial sample volume used for extraction varied from 5 to 250 mL. The majority of studies (70%) selected sample volumes between 50 and 100 mL. However, only three studies within the same group, focusing on bisphenols, utilized ≤ 20 mL as the initial sample volume [52, 66, 67]. The choice of sample volume is highly dependent on the sensitivity of the available analytical instrumentation and the specific analytes of interest. Modern instrumentation can significantly enhance sensitivity, allowing for smaller sample volumes without compromising method performance. However, the representativeness of the sample at each sampling instance remains critical, especially in the context of large-scale WWTPs. As per the reviewed literature, a median preconcentration factor of 100 was applied, with a few outliers. Notably, only two studies applied a preconcentration factor of 1000 [55, 58], which did not appear to significantly enhance overall method sensitivity, as the reported method LODs were comparable to other studies. On the other hand, Tang et al. [66, 67] achieved comparable LODs for the investigated compounds (bisphenols and phthalates) despite preconcentrating their extract by a factor of only 10. Jeong et al. [61] was the only study to mention performing a validation based on matrix effects before selecting their preconcentration factor (500 times).

#### QA/QC procedures

The validation of applied methods is crucial for ensuring accurate and reliable results in all studies, as the credibility of the findings is essential for drawing meaningful conclusions, including those in the field of WBE that offer insights into the health status of populations. While all reviewed studies were based on validated protocols, inconsistencies were observed in the reporting of method performance metrics. Although almost all studies provided information on LODs and/or LOQs, as well as the equations used for their calculation, data on matrix effects, analyte recoveries, and repeatability were notably absent. Comprehensive information on figures of merit is essential to be provided, even in the Supporting Information if previously published, for assessing method reliability and reproducibility. The validation of LC–MS/MS methodologies typically focused on accuracy, precision, linearity, sensitivity, and LODs/LOQs. However, LC-HRMS methodologies follow different approaches. Gago-Ferrero et al. [64] employed an HRMS screening method for over 2000 chemicals from various classes, which utilized a smart validation strategy based on a representative subset (10%) of compounds from the database, while the untargeted workflow utilized by Jeong et al. [61] was validated using the target compounds of the study (*N* = 35).

The QA/QC protocols that are applied in analytical methodologies enhance the reliability and transparency in the obtained results and conclusions. While most reviewed studies included dedicated paragraphs outlining the QA/QC steps they followed [55, 63], others lacked substantial information, particularly regarding the preparation of blank and spiked samples (i.e., the matrix of analysis spiked with a mix of contaminants at known concentration levels). It is recommended that all studies incorporate detailed descriptions of their QA/QC procedures, even in the supporting information files, as this is fundamental for evaluating method performance and potential contamination. The majority of the studies implemented laboratory (reagent) blank samples to evaluate potential contamination during sample preparation and instrumental analysis. Additionally, spiked samples, quality control (QC) samples, and solvent blanks were injected to investigate carryover phenomena. Jeong et al. [61] suggested a proactive approach of regularly washing the injector needle and injecting solvent blanks after each real sample injection to mitigate potential carryover issues. Implementing this practice as a standard protocol would help minimize contamination risk and improve the accuracy of the results. While analyzing field blanks can provide insights into potential contamination during sampling, only one study included this practice [66]. It is strongly recommended that future studies include field blanks as a standard QA/QC measure, especially when analyzing chemical classes prone to contamination, such as phthalates, PFAS, and bisphenols, or substances at low concentrations. The presence of these compounds in blank samples can significantly affect analyte reporting, leading to false positive results. For instance, Tang et al. [66] reported a significant variation, even up to two orders of magnitude, in the LODs for phthalates detected in blanks compared to those not detected. To address the contamination issue, additional measures should be adopted for specific chemical classes. For instance, the addition of delay columns and the use of PFAS-free liners in instruments may reduce PFAS signals in blanks. Analyte signals in blanks should be considered into the calculation of method LODs and subtracted before quantifying analytes in real samples. While some researchers merely mentioned the absence of investigated compounds in blanks or accounted for signals in blanks during reporting, others enhanced transparency by providing quantitative results for blanks, treating them as regular samples [52, 53, 60, 68]. This approach not only improves credibility of the data, but also allows for a more accurate assessment of the contamination levels and the effectiveness of the QA/QC measures.

Due to the inherent high concentrations of compounds in the investigated wastewater samples, some studies used ultrapure or tap water for preparing spiked samples for quantification and method validation [25, 48, 56]. However, the accuracy of quantification using this approach may be questionable due to differences in matrix effects. While most studies followed common clean-up and instrumental analysis procedures, the reported method LODs exhibited significant variation, ranging from 0.05 to 3000 ng L^−1^. In many cases, LODs were within the same order of magnitude for all compounds within a class. However, certain studies demonstrated LOD discrepancies exceeding 100-fold between the most and least sensitive compounds [26, 59, 66]. This variation can be attributed to differences in the compounds analyzed, the analytical protocols followed, the instrumentation used, and the equations employed for calculating LODs. The presence of analytes in blanks notably impacted the reported LODs [69]. For example, Tang et al. [66] calculated the LOQ by adding ten times the standard deviation of procedural blanks to the mean concentrations of compounds producing signals in blanks, resulting in LOQs up to two orders of magnitude higher for specific compounds.

All studies employed a mixture of isotopically labeled compounds as internal standards (IS) to monitor sample extraction efficiency, ensure proper instrumental analysis, and enable quantification using the isotope dilution method. Ideally, isotopically labeled compounds would be used for every analyzed compound, as demonstrated by Kumar et al. [51], Adhikari et al. [55], and Lopardo et al. [70]. The aforementioned studies investigated up to 16 compounds; however, most reviewed studies focused on specific classes of analytes or structurally related compounds from different classes, investigating up to 45 target compounds, while Kasprzyk-Hordern et al. [26] and Proctor et al. [69] utilized the same methodology to investigate 61 and 138 contaminants of emerging concern (CECs), respectively. Therefore, considering the high number of investigated compounds in most studies, following the 1-to-1 approach for IS and each target analyte, is limited due to the high commercial cost and restricted availability of IS. Consequently, most studies employed IS in approximately 30% of the number of target compounds they determined. To support efficient analysis and reduce costs, some vendors offer mixtures of IS in solution, particularly for compounds like PFAS. Jeong et al. [61] and Alygizakis et al. [63] conducted both targeted LC–MS/MS analysis and LC-HRMS screening methodologies, for expanding the scope of investigated compounds through untargeted approaches. Selecting IS for LC-HRMS screening methodologies poses challenges due to the wide range of compounds that are investigated. To address this, representative compounds from the target database are selected based on chemical class, structure, functional moieties, physicochemical properties, retention time, and ionization efficiency. For instance, Kasprzyk-Hordern et al. [26] spiked 30 IS for 61 CECs from different classes, while Alygizakis et al. [63] used 41 IS for both targeted analysis and untargeted workflows. Although most studies provide information on the IS used for each target compound in a table, along with instrumental parameters [58, 68, 69], some publications omit this crucial information, detracting from the credibility of the reported results.

#### Instrumental analysis

Biomarkers of exposure often occur at low concentration levels, making it essential to use analytical methodologies with high accuracy and sensitivity At low concentrations, the analyte’s signal can be close to the background noise, increasing the relative uncertainty of the measurement. High accuracy is particularly important in these cases because even minor inaccuracies can lead to significant errors in quantification. Ensuring accurate results at low concentrations is crucial for reliable exposure assessment and to avoid misinterpretation of the data. The majority of reviewed studies utilized LC–ESI–MS/MS methodologies, known for their effectiveness in determining and quantifying organic compounds in complex matrices. These methodologies offer high sensitivity and selectivity, with triple quadrupole mass analyzer and multiple reaction monitoring scan mode being the most commonly employed. Among the reviewed studies, only O’Brien et al. [59] utilized GC–MS analysis with selected ion monitoring (SIM) to determine 8 flame retardants.

WBE relies on identifying metabolites as biomarkers of exposure, because parent compounds may undergo transformation in the human body. As the chemical composition of products changes over time, there is a growing need to detect and identify both known and newly introduced biomarkers that reflect these evolving exposures. In this context, HRMS provides significant advantages over conventional targeted LC–MS/MS methods. The introduction of HRMS has revolutionized analytical instrumentation, providing accurate mass and high-resolution mass spectra, and thus enabling the precise identification and structure elucidation of a wide range of compounds, including both known and previously unknown biomarkers, with high confidence. Unlike conventional LC–MS/MS methods, which require predefined analytes, HRMS can detect and identify thousands of compounds without prior knowledge of their presence, making it invaluable for unveiling previously undetected or novel biomarkers. Following HRMS target analysis workflows, through the analysis of reference standards, can result in quantitative results, whereas for suspect and non-target screening methodologies, the development of novel semi-quantification methods [71–73] enables the estimation of concentration levels for newly identified chemicals, even when reference standards are not available, thus providing critical data for assessing population exposure to a wide range of (new) chemicals. Moreover, HRMS allows for retrospective analysis on previously acquired data, further enhancing our capacity to extract valuable exposure information. While only four studies in our review employed additional HRMS analysis, they demonstrated its potential. Lopardo et al. [70] and Kasprzyk-Hordern et al. [26] supplemented their targeted LC–MS/MS results with additional LC-QTOFMS analysis, aiming to determine BPA-sulfate and retrospectively identify and quantify specific compounds, respectively. Further processing of the already acquired data, by fully leveraging the capabilities of HRMS instrumentation, through the application of untargeted workflows, may offer additional insights into chemical exposure. Alygizakis et al. [63] conducted a suspect screening workflow by investigating 7500 known environmental contaminants and additional 1500 surfactant compounds, while Jeong et al. [61] focused on PFAS congeners using an untargeted workflow to expand their chemical investigation. The combination of LC–MS/MS and LC-HRMS techniques is highly recommended for a comprehensive investigation of population exposure to potentially harmful chemicals, as HRMS can capture a broader spectrum of compounds beyond those typically targeted in conventional LC–MS/MS methods. While HRMS offers significant advantages, its analytical capabilities may not always be fully exploited, presenting opportunities for future research objectives.

## Population normalized mass load related to harmful chemicals

After sample collection and laboratory analysis, WBE studies proceed by estimating the population normalized mass load (PNML), which represents the mass load of a chemical per inhabitant. The PNML is calculated using Eq. 1 by multiplying the concentration of the chemical in wastewater (*C*_*chemical*_, µg L^−^1) measured from a 24-h composite sample by the wastewater flow (*Q*_*wastewater*_, L d^−^1) at the sampling point on that day. This product is then divided by the number of inhabitants (*P*_*WWTP*_) discharging wastewater into the sampling point [74].

1 $$PNML \left(\mu g {d}^{-1} {inhab.}^{-1}\right)= \frac{{C}_{chemical} . {Q}_{wastewater}}{{P}_{WWTP}}$$

**Fig. 2:**
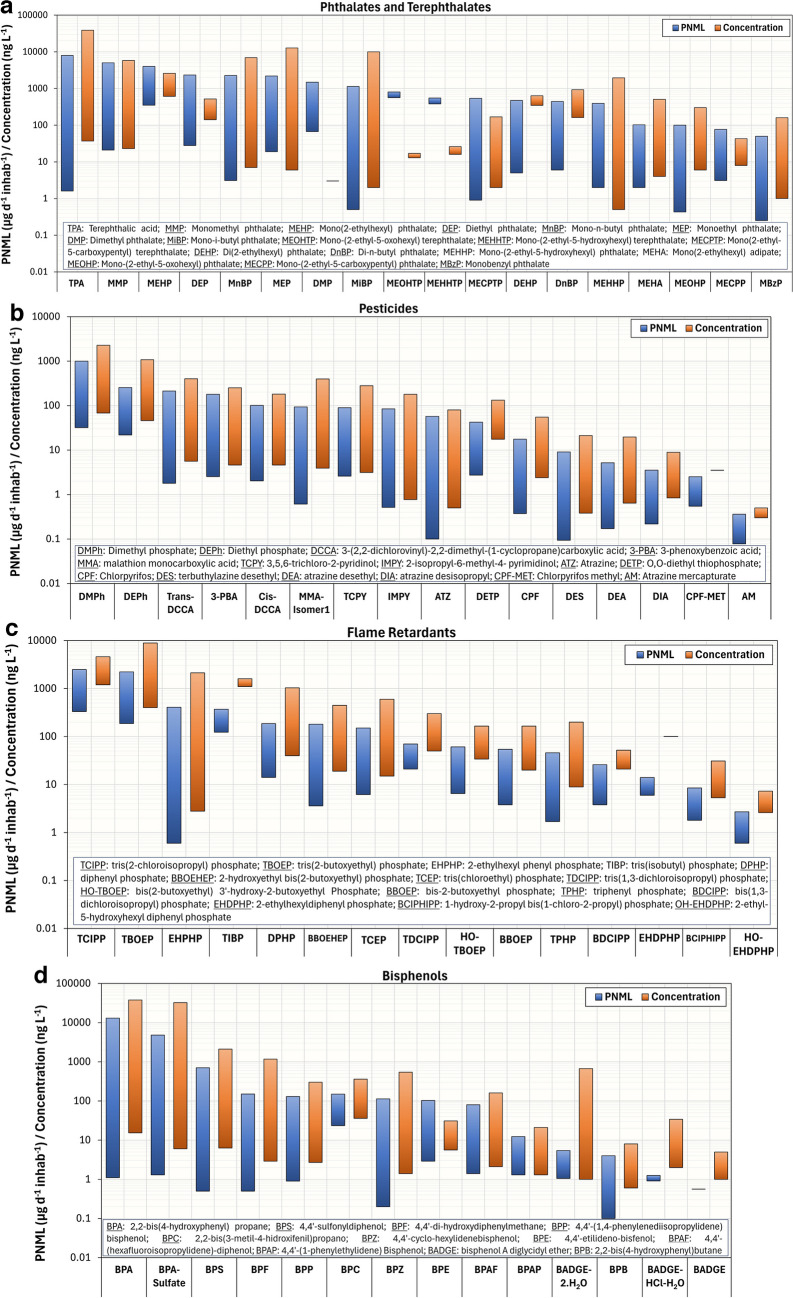
Concentration and PNML ranges of the phthalates/terephthalates (**a**), pesticides (**b**), flame retardants (**c**), bisphenols (**d**), and their metabolic products reported in WBE studies. Detailed data are shown in Tables S1–S4

**Fig. 3:**
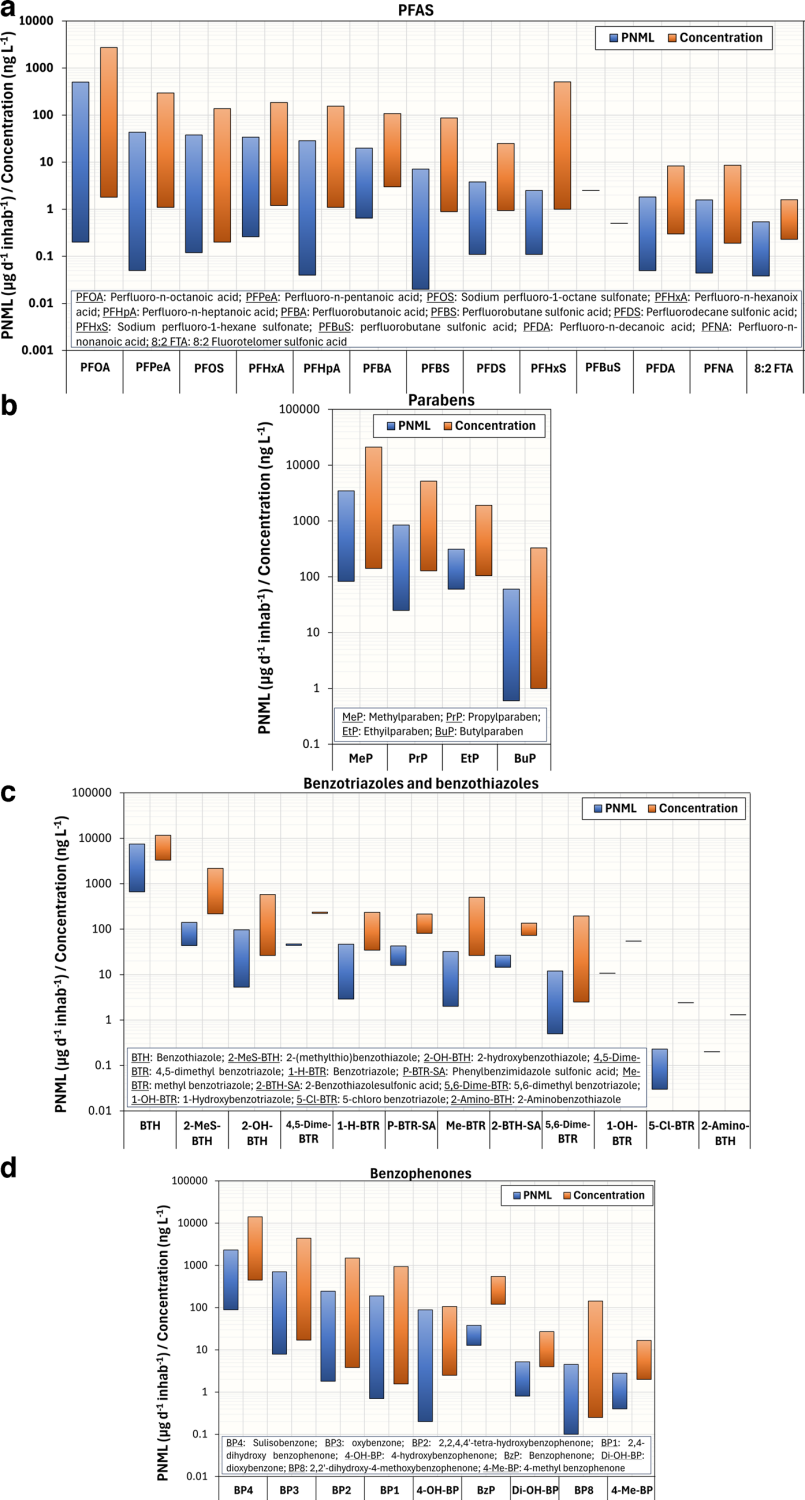
Concentration and PNML ranges of the PFAS (**a**), parabens (**b**), benzotriazoles/benzothiazoles (**c**), benzophenones (**d**), and their metabolic products reported in WBE studies. Detailed data are shown in Tables S5–S8

In this paper, we collected concentration and PNML values for harmful chemicals resulting from unintentional human exposure (Tables S1–S8), including parent compounds and their reported metabolites. We presented the calculated ranges (minimum and maximum values) in Figs. 2 and 3. The PNML values, analyzed by groups of compounds, ranged from 0.02 to 13,000 µg d^−1^ inhab^−1^ — phthalates (0.1–7967 µg d^−1^ inhab^−1^), pesticides (0.1–997 µg d^−1^ inhab^−1^), flame retardants (0.6–2490 µg d^−1^ inhab^−1^), bisphenols (0.1–13,000 µg d^−1^ inhab^−1^), PFAS (0.02–501 µg d^−1^ inhab^−1^), parabens (0.6–3459 µg d^−1^ inhab^−1^), benzotriazoles and benzothiazoles (0.2–7457 µg d^−1^ inhab^−1^), and benzophenones (0.1–2310 µg d^−1^ inhab^−1^).

Generally, PNML values for a given compound vary significantly. Bisphenols and phthalates demonstrate greater variability compared to pesticides and flame retardants. For example, BPA and its conjugate, BPA-sulfate, exhibit substantial variation—spanning four orders of magnitude. This level of variability is unexpected for compounds exclusively excreted through urine. Consequently, when studies detect large fluctuations across different locations (urban, rural, industrial) or over varying sampling periods, it may suggest the presence of additional sources beyond urine, such as industrial discharges of parent compounds. Additionally, the detection of conjugates could be associated with the transformation of parent compounds during transport through sewers, possibly mediated by biofilms. Specific arguments and citations are provided in the detailed explanations that follow for each family of compounds.

Figure 2a shows the concentration and PNML ranges reported for the family of **phthalates** and **terephthalates** compounds, and their respective metabolic products. Diester phthalates are often hydrolyzed to monoester phthalates and then excreted in urine [75, 76]. The highest concentrations detected in sewage for phthalates and terephthalates were 12.7 and 38.7 µg L^−1^ (Table S1), respectively. Some monoester phthalates showed PNML higher than 1000 µg d^−1^ inhab^−1^ — MMP (monomethyl phthalate), MEHP (mono(2-ethylhexyl) phthalate), MnBP (mono-n-butyl phthalate), MEP (monoethyl phthalate), and MiBP (mono-i-butyl phthalate). These compounds are direct derivatives from DMP (dimethyl phthalate), DEHP (di(2-ethylhexyl) phthalate), DnBP (di-n-butyl phthalate), DEP (diethyl phthalate), and DiBP (di-i-butyl phthalate), respectively. He et al. [77] found that parent phthalates are hydrolyzed in the sewers to form the monoester products. Consequently, since the source of monoester phthalates in wastewater is both the urinary excretion and the in-sewer transformation, human exposure to phthalates is overestimated by measuring monoester phthalates through WBE. The study of Tang et al. [66] found that urinary excretion was a relevant source of MEHHP (mono(2-ethyl-5-hydroxyhexyl) phthalate) (ratio of 45%) and MEOHP (mono(2-ethyl-5-oxohexyl) phthalate) (ratio of 44%) in wastewater and hence might be suitable biomarkers to assess the temporal trends of exposure (further explanations in the “WBE and its link with Human Biomonitoring” section). Regarding terephthalates and their metabolites (MEHHTP — mono-(2-ethyl-5-hydroxyhexyl) terephthalate, MEOHTP — mono-(2-ethyl-5-oxohexyl) terephthalate, MECPTP — mono(2-ethyl-5-carboxypentyl) terephthalate), TPA (terephthalic acid) was detected in a very high concentration, resulting in the highest PNML compared to its peers. However, this may be due to the presence of non-human sources, e.g., industrial effluents and the decomposition of plastic bottles in the environment. No data are available regarding the human metabolism of terephthalic acid.

**Pesticides** are one of the most widely studied harmful chemicals in the aquatic environment, with triazines, pyrethroids, and organophosphates to have been investigated more thoroughly [78–81], as it is shown in Fig. 2b, but few studies monitored the occurrence of urinary metabolites in urban wastewater. PNML levels for all compounds were found below 1000 µg d^−1^ inhab^−1^. This may be due to legal restrictions (EC nº 1107/2009, EC nº 528/2012, and REACH regulation — Registration, Evaluation, Authorization and Restriction of Chemicals) on the use of pesticides, mainly herbicides and insecticides, in the countries where the WBE studies were carried out (European Union, Switzerland, Norway, and the UK) [82]. DMPh (dimethyl phosphate) was the transformation product (TP) that presented the highest concentrations in sewage, with a maximum value of 2.3 µg L^−1^ (Table S2). This compound comes from the metabolism of several organophosphate insecticides, e.g., fosthiazate and dichlorvos. Nonetheless, the alkyl-phosphates concentrations must be examined carefully since they can also be metabolic products of other chemical classes [78, 80]. Several human biomarkers have been proposed and fully validated, since they are found to be stable in raw wastewater [79, 81]. Therefore, they can be used for estimating accurately the exposure to a specific pesticide or a group of pesticides. This means that their excretion factors were taken into account to back-calculate human exposure, instead of just calculating PNML.

Regarding the **flame retardants** (Fig. 2c), the WBE studies focused on organophosphate compounds, since their use has increased due to the ban of some brominated flame retardants [59]. TBOEP (tris(2-butoxyethyl) phosphate) and TCIPP (tris(2-chloroisopropyl) phosphate) were the compounds that presented the highest mass loads — PNML_max_ of 2230 and 2490 µg d^−1^ inhab^−1^, respectively. The maximum concentrations of these compounds (8.9 and 4.6 µg L^−1^, respectively) were much higher than their TPs (HO-TBOEP — bis(2-butoxyethyl) 3′-hydroxy-2-butoxyethyl phosphate, BBOEHEP — 2-hydroxyethyl bis(2-butoxyethyl) phosphate, BBOEP — bis-2-butoxyethyl phosphate, and BCIPHIPP — 1-hydroxy-2-propyl bis(1-chloro- 2-propyl) phosphate), which highlights the influence of other sources of contamination (apart from human excretion) [65]. The TP of organophosphorus flame retardants (OPFRs) that was found in the greatest proportion in sewage was EHPHP (2-ethylhexyl phenyl phosphate) — maximum concentration of 2.1 µg L^−1^. It is known to be formed from EHDPHP (2-ethylhexyl diphenyl phosphate), the main OPFR found in food samples, which presented negligible concentrations in sewage (Table S3) [56, 57]. Thus, EHPHP constitutes the main biomarker of exposure of human metabolism to OPFRs, although investigation should be also performed on the transformation of EHDPHP via other processes, because no data are available in the literature.

The concentrations of **bisphenol** analogues (bisphenol-B, C, E, F, P, S, AF, AP) have increased in different environmental matrices mainly due to the limitations of the use of bisphenol-A (BPA) resulting from its adverse effects on human health [83]. Nonetheless, studies have shown that these analogues exhibit potential estrogenic activities and similar or even stronger toxic effects than BPA [52]. BPA concentrations in sewage and its mass loads continue to be higher than their analogues, as it is shown in Fig. 2d, due to the diversity of potential sources of human exposure — food, drinking water, personal care products, and contaminated air [84]. Very significant concentrations in sewage and PNML values of BPA-sulfate were reported (32.4 µg L^−1^ and 4800 µg d^−1^ inhab^−1^, respectively) (Table S4). However, based on the results of human biomonitoring studies (urinary BPA concentration) [85] and pharmacokinetic data (excretion fraction of BPA-sulfate) [86], these PNML values are higher than the expected, suggesting that there are additional sources of BPA-sulfate in wastewater, which limits its use as human biomarker in WBE studies.

Figure 3a shows the concentration and PNML ranges reported for the family of **PFAS (**per- and polyfluoroalkyl substances). Although there are several sources of human exposure to PFAS, few studies report their concentrations associated with mass load levels per capita background [61–63]. The highest concentrations and PNML values reported were those related to PFOA (perfluorooctanoic acid) (concentration = 2.7 µg L^−1^, PNML = 501 µg d^−1^ inhab^−1^) (Table S5), the most well-known perfluoroalkyl carboxylic acid. Among the investigated compounds, no known PFAS metabolites were detected in sewage, which might be related to human internal metabolism. Indeed, Nguyen et al. [62] evidenced that the per capita background release of PFAS to wastewater treatment plants is mainly due to contributions from catchment specific point sources (i.e., industry, airports, military bases, and landfills), extrapolating the PNML values. Consequently, detected in wastewater PFAS cannot be proposed as human biomarkers of exposure.

Similarly, for **parabens**, no TPs potentially related to human exposure were detected in wastewater (Fig. 3b), and their PNML values might be related to various sources of contamination in sewage or the use of many personal care products. Senta et al. [65] investigated the presence of known human metabolites of methylparaben and ethylparaben, which were the compounds methyl 3,4-dihydroxybenzoate (3-OH-MeP) and ethyl 3,4-dihydroxybenzoate (3-OH-EtP), respectively, but they were not detected in sewage. Methylparaben was detected in a quite high concentration in sewage (21.1 µg L^−1^), with its associated PNML (3,459 µg d^−1^ inhab^−1^) (Table S6) [69], likely linked to the intensive use of cosmetics and personal care products [55]. Since no metabolic products were investigated in this study, the high PNML of methylparaben could come from direct disposal of products and waste from the respective industries, but, in any case, its presence in wastewater is correlated to the high production and thus potential use of cosmetics and personal care products including this chemical.

Regarding the class of **benzotriazoles** (BTRs) and **benzothiazoles** (BTHs), Fig. 3c shows higher levels of human exposure in the group of BTHs, with concentrations of 11.6 µg L^−1^ and PNML of 7457 µg d^−1^ inhab^−1^ [48]. The main chemicals from benzothiazoles’ family were 2-OH-BTH (2-hydroxybenzothiazole) (concentration_max_ = 0.6 µg L^−1^, PNML_max_ = 97 µg d^−1^ inhab^−1^) and 2-MeS BTH (2-(methylthio)benzothiazole) (concentration_max_ = 2.2 µg L^−1^, PNML_max_ = 141 µg d^−1^ inhab^−1^) (Table S7). Since the compounds benzotriazole (1-H-BTR) and benzothiazole (BTH) presented higher mass loads compared to their TPs, it is also concluded that the concentrations of these chemicals in sewage are due to diffuse sources of contamination. For example, 1-H-BTR is used as flame inhibitors, deicing and antiicing agent, pigments, and dishwasher detergents, while BTH is used as tire additives, corrosion inhibitors, and antifogging agents [39].

The occurrence of **benzophenone**-type ultra-violet (UV) light filters, especially 2-hydroxy-4-methoxy benzophenone (BP-3), in sewage is frequently reported, due to their presence and use in conditioners, cosmetics, and sunscreen lotions, to protect skin against harmful UV radiation [60, 63]. The highest PNML values were referring to the compounds BP-3 and BP-4 (2-hydroxy-4-methoxybenzophenone-5-sulfonic acid) (Fig. 3d), probably due to the fact that among the benzophenones, only these chemicals are allowed in cosmetics by the European Legislation [87]. BP-4 was present in higher concentrations and PNML (concentration_max_ = 14.1 µg L^−1^, PNML_max_ = 2310 µg d^−1^ inhab^−1^) (Table S8).

## WBE and its link with human biomonitoring

A key challenge in using WBE to assess unintentional human exposure to potentially harmful chemicals is the need to identify specific exposure biomarkers that originate exclusively from human excretion. Without confirming the unique human origin of the detected chemicals, their presence in wastewater may simply reflect their widespread production, use in consumer products, or direct release into the environment. This would imply only potential indirect effects on human health rather than providing direct evidence of exposure through their presence in the human body. The limited availability of studies containing experimental or computational metabolism data on humans (or animals), along with data on stability, in-sewer transformation, and sorption of these chemicals in the environment, adds complexity to validating WBE as a reliable tool for characterizing unintentional human exposure, particularly for certain chemical families. Despite the difficulty in correlating wastewater concentrations with human exposure levels and potential health risks, the true innovation in expanding WBE lies in its integration with HBM studies, and ideally, in vitro assays.

Specifically, HBM is crucial for enhancing confidence in WBE results, as it aids in determining suitable and unsuitable biomarkers for exposure assessment. A common practice to ascertain whether a biomarker originates solely from urine, and thus qualifies as an appropriate biomarker of exposure, is to compare the concentrations of the biomarkers measured in urine samples to the estimated concentrations in urine through the PNML and using Eq. 2. In this equation, CF is the correction factor calculated by using Eq. 3. 1.57 is the volume (in L) of urine per person and day provided by González-Mariño et al. [54]

2 $${Urine}_{concentration} \left(\mu g {L}^{-1}\right)= \frac{PNML . \text{CF}}{1.57}$$

3 $$CF=\frac{Molecular\;weight\;parent}{Molecular\;weight\;metabolite.Molar\;excretion\;ratio}$$

Calculating the urinary contribution of a biomarker to wastewater (%) could indicate the concentration of the biomarker in wastewater that may be due to human excretion from the population. This practice proves valuable, especially when data on chemical metabolism, excretion, in-sewer transformation processes, and stability are limited. To be effectively implemented, careful planning of integrated WBE-HBM studies is valuable, ensuring that urine and wastewater samples are collected from the same location and time period. This minimizes uncertainty regarding the exposure and the source of the chemicals, whether from human excretion or other origins. However, such comparative analyses are rarely conducted using samples collected within the same time and area, highlighting the need for further verification.

Examples of such comparisons can be found in literature. In a study focusing on bisphenols [67], the urinary contribution of BPA to wastewater was 0.81%, while that of bisphenol S (BPS) 0.16%. This indicates that human intakes of BPA are 1–2 orders of magnitude lower than those estimated from WBE, suggesting that urinary excretion is not a major source for the BPA and BPS in wastewater in South East Queensland. Instead, other sources, such as direct leaching from plastic products and industrial inputs, are likely the primary contributors of bisphenols reaching WWTPs. Similarly, Wang et al. [52] reported ratios below 5% for BPA, BPS, and bisphenol AF (BPAF), highlighting the inadequacy of relying solely on parent compound concentrations for assessing unintentional human exposure to chemicals. A study on phthalates, which included metabolites [66], reported that the urinary excretion of phthalate metabolites accounted for between 0.33% (MMP) and 45% (MEHHP) of the mass loads in wastewater. Urinary excretion was found to be a relevant source of MEHHP (ratio of 45%) and MEOHP (ratio of 44%) in wastewater. However, for most analytes, the ratios were lower than 25%, i.e., MMP (0.33%), MiBP (5.4%), MBP (9.7%), MBzP (20%), and MEP (23%), suggesting that for most phthalate urinary excretion of these chemicals, the population is unlikely the main source in the wastewater.

The study from He et al. [77] concluded that phthalate metabolites can be strongly influenced by in-sewer transformation of the parent phthalates and of themselves, and could not be assumed as uniquely the results of urinary excretion after human exposure to parent phthalates. Hence, not only stability tests need to be conducted for metabolites but also in-sewer transformation studies to conclude whether a metabolite is a suitable biomarker of exposure or not. We suggest that for biomarkers for which the urinary excretion contribution to sewer is significant (e.g., > 40%), such compounds can be used to assess the temporal trends of human exposure. However, estimating exposure is highly risky without thorough validation of in-sewer transformations and stability tests [77]. Care must be taken when conducting such tests in biofilm-free reactors. The study by Choi et al. [88] compared the stability of 32 pharmaceutical and personal care products (PPCPs) in biofilm-free reactors with that in-sewer lab reactors, and compared with literature outcomes from pilot and actual sewers. They concluded that biofilm-free studies underestimate the degradation of biomarkers.

On the other hand, WBE can serve as a powerful complementary tool to HBM, thereby enhancing its effectiveness and potential. Firstly, because of its capacity to objectively track the population’s unintentional exposure to certain chemicals at a low cost. WBE can be used to assess which biomarkers accumulate in humans and may be potentially harmful, serving as an early warning system for detecting chronic exposures that could be damaging in the long term, thereby allowing for earlier intervention. This has been approached by measuring the concentration of biomarkers in primary sludge at WWTPs in the study of Gil-Solsona et al. [6]. This study collected and analyzed biospecimens from pregnant women, such as maternal blood and placenta during labor, alongside sewage sludge from a WWTP serving hospitals and their residential areas during the same period. Advanced software and tools were utilized to process the data, qualitatively define the presence of xenobiotics, and assess their (semi)quantitative levels, facilitating the correlation of findings across different matrices. An overlap was observed in the identified chemicals and unknown features (which may also include endogenous chemicals) between the biospecimens and sewage sludge. The authors suggested that sewage sludge, which is rich in carbon and lipids, could serve as an indicator of possible bioaccumulation of xenobiotics in humans, because, as stated in detail elsewhere [89], the mechanism of partitioning of hydrophobic compounds to sewage sludge is similar to the way these chemicals bioaccumulate in organisms. Hence, substances found in sewage sludge present at high concentration, high frequency of detection or being potentially toxic, are worthy of inclusion in HBM studies.

Selection of appropriate biomarkers is pivotal in environmental exposure assessment. While human metabolites often provide more accurate indicators of exposure compared to parent compounds, determining which ones to focus on can be challenging. HBM is resource-intensive, prompting exploration of alternatives such as in vitro assays. Alternatively, in vitro assays offer a complementary approach to HBM for identifying metabolic products. Lopardo et al. [90] proposed a workflow utilizing pooled human liver microsomes (HLM) to identify metabolites of 4-chloro-m-cresol in wastewater and urine samples. This approach facilitated the identification of metabolites indicative of unintentional exposure, bridging the gap between wastewater analysis and human biomonitoring.

Overall, meticulous attention is essential when utilizing WBE to estimate unintentional exposures, with various factors requiring consideration. Neglecting parameters such as metabolism data, in-sewer transformations and stability, and direct industrial disposal can compromise WBE’s ability to identify specific biomarkers of unintentional exposure accurately. However, when WBE is coupled with HBM, it furnishes more reliable data regarding direct chemical exposure through ingestion, inhalation, and dermal contact. Moreover, WBE provides an avenue to evaluate the efficacy of measures aimed at reducing emissions and chemical exposure with relative ease [24]. By furnishing comprehensive data on multi-chemical exposure among urban residents while maintaining anonymity [91, 92], WBE has the potential to significantly contribute to epidemiological research. Finally, more studies linking WBE and HBM are needed, collecting wastewater samples and human specimens from the same population sampled at similar spatial and temporal scales. Such an approach would reduce uncertainties and shed more light on the selection of appropriate biomarkers.

## Supplementary Information

Below is the link to the electronic supplementary material.

Supplementary file1 (PDF 574 KB)
