# Supplementary material for: A critical review of wastewater-based epidemiology as a tool to evaluate the unintentional human exposure to potentially harmful chemicals
Source: Anal Bioanal Chem. 2024 Oct 18;417(3):495–511. doi: 10.1007/s00216-024-05596-z (PMC11700037; doi:10.1007/s00216-024-05596-z)
Supplement: Supplementary file 1 — Supplementary file1 (PDF 574 KB) [file 216_2024_5596_MOESM1_ESM.pdf]

Supporting Information

A critical review on wastewater-based epidemiology as a tool to evaluate the unintentional human exposure to potentially harmful chemicals

Rodrigo B. Carneiro[a*], Maria-Christina Nika[b], Rubén Gil-Solsona[c], Konstantina S. Diamanti[c], Nikolaos S. Thomaidis[c], Lluís Corominas[d,e], Pablo Gago-Ferrero[b**]

[a] Laboratory of Chromatography, São Carlos Institute of Chemistry (IQSC), University of São Paulo (USP), 400, Trabalhador São-Carlense Ave., São Carlos, São Paulo 13566-590, Brazil

e-mail: rodrigocarneiro@sc.usp.br

[b] Department of Environmental Chemistry, Institute of Environmental Assessment and Water Research (IDAEA), Severo Ochoa Excellence Center, Spanish Council of Scientific Research (CSIC), Jordi Girona 18-26, E-08034, Barcelona, Spain

e-mail: mariachristina.nika@idaea.csic.es; pablo.gago@idaea.csic.es

[c] Laboratory of Analytical Chemistry, Department of Chemistry, National and Kapodistrian University of Athens, Panepistimiopolis Zografou, 15771, Athens, Greece

e-mail: rgilsolsona@chem.uoa.gr; kdiamanti@chem.uoa.gr; ntho@chem.uoa.gr

[d] Catalan Institute for Water Research (ICRA-CERCA), Emili Grahit 101, 17003 Girona, Catalonia, Spain

e-mail: lcorominas@icra.cat

[e] University of Girona, Plaça de Sant Domènec 3, 17004 Girona, Catalonia, Spain

Corresponding authors:

* rodrigocarneiro@sc.usp.br, Phone: +55 (16) 3373-8357 / 3373-8358;

** pablo.gago@idaea.csic.es; Phone: +34 934006100

Table S1. Concentration and PNML ranges of the phthalates / terephthalates and their metabolites reported in WBE studies.

**Parent Compound**	**Metabolite**	**Acronym**	**Concentration Range (ng L^{-1})**		**PNML Range (µg d^{-1} $inhab^{-1}$)**		**Ref.**
			**Min**	**Max**	**Min**	**Max**	
Dimethyl phthalate (DMP)	Monomethyl phthalate	MMP	48	1885	32	958	[1]
			226	714	131	222	[2]
			72	3828	42	717	[3]
			119	868	NA*	36	[4]
			860	5800	550	5000	[5]
			23	2670	21	284	[6]
		DMP	NA	NA	67	1488	[1]
			NA	NA	117	389	[3]
			NA	3	NA	0.5	[7]
Diethyl phthalate (DEP)	Monoethyl phthalate	MEP	300	1599	164	1419	[1]
			100	820	19	141	[4]
			335	12700	138	1465	[3]
			1200	3100	450	600	[5]
			6	1581	40	567	[6]
			2656	3690	1453	2190	[2]
			113	2040	66	460	[8]
		DEP	NA	NA	336	2341	[1]
			NA	NA	181	2002	[3]
			141	521	28	103	[7]
Di-i-butyl phthalate (DiBP)	Mono-i-butyl phthalate	MiBP	67	277	43	224	[1]
			550	3900	450	800	[5]
			39	1974	11	188	[3]
			2	2599	0.5	1132	[6]
			223	334	121	196	[2]
			33	416	19	104	[8]
Di-n-butyl phthalate (DnBP)	Mono-n-butyl phthalate	MnBP	55	274	43	224	[1]
			7	867	3.1	131	[3]
			93	6921	204	2263	[6]
			219	795	139	484	[2]
			17	480	10	120	[8]
		DnBP	NA	NA	54	392	[1]
			NA	NA	6	441	[3]
			162	925	32	184	[7]
Butyl benzyl phthalate (BzBP)	Monobenzyl phthalate	MBzP	1	19	0.25	17	[1]
			20	160	10	50	[5]

			90	138	NA	25	[4]
			2	31	1	7	[6]
			15	23	8.3	13	[2]
		BzBP	NA	NA	0.12	28	[1]
			NA	NA	0.21	12	[3]
Di(2-ethylhexyl) phthalate (DEHP)	Mono(2-ethylhexyl) phthalate	MEHP	610	2600	350	4000	[5]
	Mono-(2-ethyl-5-hydroxyhexyl) phthalate	MEHHP	11	62	4.3	56.2	[1]
			203	1952	NA	393	[4]
			67	270	25	50	[5]
			18	170	0.54	27	[3]
			0.5	102	18	178	[6]
			18	27	51	80	[2]
			5	18	2	5	[8]
	Mono-(2-ethyl-5-oxohexyl) phthalate	MEOHP	6	84	0.9	32.6	[1]
			40	300	20	60	[5]
			10	128	0.43	20	[3]
			60	179	NA	26	[4]
			14	24	56	100	[2]
	Mono-(2-ethyl-5-carboxypentyl) phthalate	MECPP	8	43	3.1	39	[1]
			19	26	55	77	[2]
		DEHP	NA	NA	35	472	[1]
			NA	NA	5	90	[3]
			347	633	68	126	[7]
Polyethylene terephthalate	Terephthalic acid	TPA	37	38653	1.6	7967	[4]
Di(2-ethylhexyl) terephthalate (DEHTP)	Mono-(2-ethyl-5-hydroxyhexyl) terephthalate	MEHHTP	16	26	381	551	[2]
	Mono-(2-ethyl-5-oxohexyl) terephthalate	MEOHTP	13	17	561	802	[2]
	Mono(2-ethyl-5-carboxypentyl) terephthalate	MECPTP	125	169	422	541	[2]
			2	116	0.9	23	[8]
Bis(2-ethylhexyl) adipate (DEHA)	Mono(2-ethylhexyl) adipate	MEHA	4	508	2	102	[8]

* NA – Not available

Table S2. Concentration and PNML ranges of the pesticides and their metabolites reported in WBE studies.

Parent Compound	Metabolite	Acronym	Concentration Range (ng L^{-1})		PNML Range (µg d^{-1} $inhab^{-1}$)		Ref.
			Min	Max	Min	Max	
Atrazine		ATZ	1	29.4	0.10	6.30	[9]
			1.3	12.8	0.33	3.38	[10]
			0.5	80	0.16	57.25	[11]
			1	2.7	NA*	1.00	[12]
			1.2	19	0.26	4.81	[13]
Terbuthylazine	Terbuthylazine desethyl	DES	0.6	21.1	0.09	4.97	[10]
			1.7	19.2	0.40	9.04	[11]
			0.38	1.9	NA	0.40	[12]
			2.6	13.2	0.59	4.16	[13]
			1.05	1.83	0.10	0.40	[9]
Atrazine	Atrazine desisopropyl	DIA	1.4	8.9	0.22	3.54	[10]
			1.4	6.6	0.33	2.22	[11]
			0.84	5.2	NA	1.20	[12]
			1.4	4.1	0.30	1.82	[13]
Atrazine	Atrazine desethyl	DEA	1.1	19.6	0.17	5.17	[10]
			1.1	8.5	0.26	3.72	[11]
			0.64	5	NA	1.40	[12]
			5.1	19.6	1.55	4.44	[13]
Atrazine	Atrazine mercapturate	AM	0.5	0.5	0.08	0.24	[10]
			0.5	0.5	0.12	0.36	[11]
			0.5	0.5	0.11	0.22	[13]
Various Pyrethroids	3-phenoxybenzoic acid	3PBA	5.3	129	2.52	30.37	[10]
			38	181	11.76	129.54	[11]
			16.5	251.1	5.55	179.71	[14]
			9.2	191	4.09	43.22	[13]
			4.6	12.4	NA	4.10	[12]
			174	212	20.40	43.50	[9]
trans-permethrin, −cypermethrin, −cyfluthrin	3-(2,2-dichlorovinyl)-2,2-dimethyl-(1-cyclopropane)carboxylic acid	trans-DCCA	15.1	200	7.19	47.08	[10]
			27	224	8.64	160.31	[11]
			5.6	297.7	1.79	213.06	[14]
			11	129.2	3.30	33.70	[12]
			30.5	401	12.75	90.74	[13]
			112	301	25.90	62.30	[9]
cis-permethrin, −cypermethrin, −cyfluthrin	3-(2,2-dichlorovinyl)-2,2-dimethyl-(1-cyclopropane)carboxylic acid	cis-DCCA	7.7	45	2.03	10.59	[10]
			7.7	86	2.46	61.55	[11]
			7.7	140.8	2.59	100.77	[14]
			7.7	181	3.42	40.96	[13]
			10	112	2.10	12.30	[9]
Chlorpyrifos		CPF	2.4	2.4	0.37	1.14	[10]
			2.4	55	0.56	17.60	[11]
			2.4	2.4	0.52	1.07	[13]
Chlorpyrifos	Chlorpyrifos methyl	CPF-MET	3.5	3.5	0.55	1.67	[10]
			3.5	3.5	0.82	2.50	[11]
			3.5	3.5	0.75	1.55	[13]

Chlorpyrifos, chlorpyrifos-methyl	3,5,6-trichloro-2-pyridinol	TCPY	8.3	93	3.95	21.89	[10]
			12	280	4.56	89.60	[11]
			3.13	11.7	2.60	3.90	[12]
			15.9	162	5.54	36.66	[13]
Malathion	Malathion monocarboxylic acid	MMAisomer1	3.9	397	0.61	93.45	[10]
			3.9	43	1.25	30.77	[11]
			3.9	13.2	0.84	4.16	[13]
		MMAisomer2	4.8	285	0.75	67.09	[10]
			4.8	53	1.54	37.93	[11]
			4.8	9.6	1.03	3.02	[13]
Diazinon	2-isopropyl-6-methyl-4-pyrimidinol	IMPY	1.29	72	0.51	16.99	[10]
			1.8	179.2	0.68	84.33	[11]
			0.77	80.1	NA	19.30	[12]
			1.8	137	0.57	31.00	[13]
			5.1	8.4	0.60	1.80	[9]
Several organophosphate insecticides	Diethyl phosphate	DEPh	46	1076	21.91	253.88	[10]
			88	717	33.44	229.44	[11]
			62	117	27.00	31.00	[12]
			250	296	63.33	131.48	[13]
			224	380	30.44	63.50	[9]
	O,O-diethyl thiophosphate	DETP	17.5	39	2.73	9.20	[10]
			17.5	132	4.12	42.24	[11]
			17.5	17.5	4.43	7.77	[13]
			35	114	5.53	12.44	[9]
	Dimethyl phosphate	DMPh	128	2269	41.91	996.63	[10]
			105	1686	42.18	539.52	[11]
			68	460	32.00	133.00	[12]
			252	427	68.91	189.67	[13]
			277	393	34.36	78.60	[9]

* NA – Not available

Table S3. Concentration and PNML ranges of the flame retardants and their metabolites reported in WBE studies.

Parent	Metabolite	Acronym	Concentration Range (ng L^{-1})		PNML Range (µg d^{-1} hab^{-1})		Ref
			Min	Max	Min	Max	
2-ethylhexyldiphenyl phosphate	2-ethyl-5-hydroxyhexyl diphenyl phosphate	HO-EHDPHP	2.6	7.3	0.6	2.7	[15]
			2.9	6.5	0.6	2	[16]
	2-ethylhexyl phenyl phosphate	EHPHP	168	1100	38	396	[15]
			284	2124	84.2	407.8	[16]
			2.8	14	0.6	4.4	[8]
	diphenyl phosphate	DPHP	71	628	17	175	[15]
			86	1037	31.4	186	[16]
			40	195	14.1	48.8	[8]
		EHDPHP	<100	100	6	14	[17]
tris(2-butoxyethyl) phosphate	bis(2-butoxyethyl) 3′-hydroxy-2-butoxyethyl phosphate	HO-TBOEP	43	165	12	61	[15]
			34	112	6.5	47.3	[16]
	2-hydroxyethyl bis(2-butoxyethyl) phosphate	BBOEHEP	24	94	6.9	35	[15]
			19	108	3.6	29.7	[16]
			48	447	20	181	[8]
	bis-2-butoxyethyl phosphate	BBOEP	20	165	3.8	54.3	[16]
		TBOEP	400	6600	187	1778	[17]
			1951	8901	578	2228	[8]
tris(2-chloroisopropyl) phosphate	1-hydroxy-2-propyl bis(1-chloro-2-propyl) phosphate	BCIPHIPP	5.3	8.9	1.8	3.3	[15]
			15	31	2.7	8.5	[16]
		TCIPP	1200	4100	436	902	[17]
			1273	4582	333	2490	[8]
triphenyl phosphate		TPHP	<200	200	46	46	[17]
			19	119	7.8	30	[8]
			8.9	12.6	1.7	2.5	[7]
tris(chloroethyl) phosphate		TCEP	211	389	50	108	[15]
			213	323	38.2	128.7	[16]
			200	600	35	151	[17]
			15	275	6.2	77.9	[8]
tris(1,3-dichloroisopropyl) phosphate	bis(1,3-dichloroisopropyl) phosphate	BDCIPP	21	52	3.8	26	[16]
		TDCIPP	50	300	21	70	[17]
Tris(isobutyl) phosphate		TIBP	1100	1600	123	371	[17]

Table S4. Concentration and PNML ranges of the bisphenols and their metabolites reported in WBE studies.

Parent	Metabolite	Acronym	Concentration Range (ng L^{-1})		PNML Range (μg d^{-1} hab^{-1})		Ref
			Min	Max	Min	Max	
Bisphenol-A		BPA	15.4	184.5	1.1	30.8	[18]
			230	5200	97	2184	[19]
			100	6200	200	7400	[20]
			<30	8550	24.9	75	[21]
			480	32000	170	13000	[22]
			<58	123	NA	47	[4]
			748	37729	163	6659	[23]
			437	484	87	95	[7]
Bisphenol-A	Bisphenol-A Sulphate	BPA - sulphate	2700	32400	400	4800	[20]
			6	45	7	19.8	[8]
			<59	65	NA*	13	[4]
Bisphenol-S		BPS	6.7	32.3	0.5	5.4	[18]
			90	1100	46	340	[19]
			<20	301	12.9	26	[21]
			500	2100	190	440	[5]
			564	1207	118	702	[8]
			102	722	20	150	[4]
			6.3	10.1	1.2	2	[7]
Bisphenol-F		BPF	2.9	27.6	0.5	1.8	[18]
			<1.4	180	NA	150	[19]
			<100	1170	75.1	75.1	[21]
Bisphenol-AF		BPAF	NA	2.8	NA	0.4	[18]
			6.6	160	1.4	80	[19]
Bisphenol-AP		BPAP	<1.3	1.7	NA	0.1	[18]
			<13	21	NA	11	[19]
Bisphenol-P		BPP	NA	26.6	NA	4.4	[18]
			2.7	300	0.9	129	[19]
Bisphenol-Z		BPZ	NA	3.2	NA	0.2	[18]
			<1.4	540	NA	113	[19]
Bisphenol-B		BPB	0.6	5.5	0.1	0.7	[18]
			<2.1	8	NA	4	[19]
Bisphenol-C		BPC	<36	360	NA	126	[19]
Bisphenol-E		BPE	<5.6	31	NA	100	[19]
Bisphenol-A	Bisphenol A diglycidyl ether	BADGE	<1	4.95	0.56	0.56	[21]
Bisphenol-A	Bisphenol A diglycidyl ether	BADGE·2H2O	<1	673	1.05	5.4	[21]
Bisphenol-A	bisphenol A diglycidyl ether	BADGE·HCl·H2O	<2	34	0.91	1.25	[21]

Table S5. Concentration and PNML ranges of the PFAS reported in WBE studies.

Parent / Metabolite	Acronym	Concentration Range (ng L^{-1})		PNML Range (µg d^{-1} hab^{-1})		Ref
		Min	Max	Min	Max	
Perfluorobutane sulfonic acid	PFBS	2.5	23	0.56	7.15	[24]
		0.89	87	0.02	4.60	[25]
Sodium perfluoro-1-hexane sulfonate	PFHxS	1	9	0.27	1.88	[24]
		1.3	509	0.11	2.50	[25]
Sodium perfluoro-1-octane sulfonate	PFOS	2.8	101	0.40	20.29	[24]
		1.1	137	0.12	2.90	[25]
		0.2	7.5	1.30	37.70	[7]
Perfluorobutanoic acid	PFBA	3	108	0.65	19.86	[24]
perfluorobutane sulfonic acid	PFBuS	NA*	0.5	NA	2.50	[7]
Perfluoro-n-pentanoic acid	PFPeA	3.4	295	0.92	43.31	[24]
		1.1	31	0.05	1.70	[25]
Perfluoro-n-hexanoix acid	PFHxA	2.7	185	0.48	34.03	[24]
		2.1	119	0.26	2.40	[25]
		1.2	3	6.30	15.10	[7]
Perfluoro-n-heptanoic acid	PFHpA	2.5	155	0.47	28.51	[24]
		1.1	17	0.04	1.50	[25]
		NA	0.2	NA	1.30	[7]
Perfluoro-n-octanoic acid	PFOA	2.4	2726	0.35	501.37	[24]
		1.8	33	0.20	3.30	[25]
		11.4	22.6	2.20	4.50	[7]
Perfluoro-n-nonanoic acid	PFNA	0.19	8.6	0.04	1.58	[24]
Perfluoro-n-decanoic acid	PFDA	0.3	8.3	0.05	1.83	[24]
8:2 Fluorotelomer sulfonic acid	8:2 FTS	0.23	1.6	0.04	0.54	[24]

* NA – Not available

Table S6. Concentration and PNML ranges of the parabens reported in WBE studies.

Parent	Acronym	Concentration Range (ng L^{-1})		PNML Range (µg d^{-1} hab^{-1})		Ref
		Min	Max	Min	Max	
Methylparaben	MeP	142	2819	83	569	[8]
		2584	21135	512	3459	[23]
		NA*	NA	300	1783	[27]
Ethylparaben	EtP	105	591	61	307	[8]
		487	1915	111	313	[23]
		NA	NA	60	250	[27]
Propylparaben	PrP	128	840	74	194	[8]
		847	5184	192	848	[23]
		NA	NA	25	225	[27]
Butylparaben	BuP	1	28	0.6	6.6	[8]
		59	329	13	54	[23]
		NA	NA	5	60	[27]

* NA – Not available

Table S7. Concentration and PNML ranges of the benzotriazoles / benzothiazoles reported in WBE studies.

Parent	Metabolite	Acronym	Concentration Range (ng L^{-1})		PNML Range (µg d^{-1} hab^{-1})		Ref
			Min	Max	Min	Max	
Benzotriazole		BTR	34.5	167	2.9	9.1	[18]
			154.7	234	30.4	46.6	[7]
Benzotriazole	Methyl benzotriazole	Me-BTR	26.4	503	2	32.3	[18]
Benzotriazole	5,6-dimethyl benzotriazole	5,6-DiMe-BTR	5.6	195	1.1	12	[18]
			2.5	10.1	0.5	2	[7]
Benzotriazole	5-chloro benzotriazole	5-Cl-BTR	NA*	2.4	NA	0.2	[18]
Benzotriazole	Benzotriazole -4-Me;Benzotriazole- 5-Me	4,5-DiMe-BTR	222	237	44	47	[7]
Benzotriazole	Benzotriazole-1-Hydroxy	1-OH-BTR	54.5	54.5	10.7	10.7	[7]
Benzotriazole	Phenylbenzimidazole sulfonic acid	P-BTR-SA	81	215	16	42.8	[7]
Benzothiazole		BTH	3310	11600	662	7457.1	[18]
Benzothiazole	2-hydroxybenzothiazole	2-OH-BTH	293	579	19.2	96.5	[18]
Benzothiazole			26.4	76.1	5.3	15	[7]
Benzothiazole	2-(methylthio)benzothiazole	2-MeS BTH	217	2185	43.4	140.5	[18]
Benzothiazole	Benzothiazole- 2-Amino	2-Amino-BTH	1.3	1.3	0.2	0.2	[7]
Benzothiazole	2-Benzothiazolesulfonic acid	2-BTH-SA	73	136	14.5	26.7	[7]

* NA – Not available

Table S8. Concentration and PNML ranges of the benzophenones and their metabolites reported in WBE studies.

Parent	Metabolite	Acronym	Concentration Range (ng L^{-1})		PNML Range (μg d^{-1} hab^{-1})		Ref
			Min	Max	Min	Max	
Benzophenone		BzP	120	548	12.8	38	[18]
Benzophenone		BP3	17	295	7.9	245.7	[26]
			31	288	12.8	58.1	[8]
			635	4392	144	703	[23]
			396	718	77.8	142.9	[7]
Benzophenone-3	2,4-dihydroxy benzophenone	BP1	19.8	113	1.5	7.7	[18]
			1.56	122	0.7	38.5	[26]
			38	937	16	189	[8]
			112	862	24.4	141	[23]
Benzophenone-3	2,2,4,4'-tetra-hydroxybenzophenone	BP2	3.81	123	1.8	57.4	[26]
			26	1490	5.7	243.8	[23]
Benzophenone-3	2-Hydroxy-4-methoxybenzophenone-5-sulfonic acid	BP4	2792	14116	610	2310	[23]
			448	620	89.1	122	[7]
Benzophenone-3	2,2'-dihydroxy-4-methoxybenzophenone	BP8	1.7	12.1	0.1	2	[18]
			0.25	143	0.1	4.5	[26]
Benzophenone-3	4-hydroxybenzo phenone	4-OH-BP	2.5	21.4	0.2	2.4	[8]
			15.7	106	7.3	88.3	[23]
Benzophenone	4-methyl benzophenone	4-MeBP	2	16.7	0.4	2.8	[18]
Benzophenone	Dioxybenzone	Di-OH-BP	4	27	0.8	5.2	[7]

References

1. González-Mariño I, Rodil R, Barrio I, Cela R, Quintana JB (2017) Wastewater-Based Epidemiology as a New Tool for Estimating Population Exposure to Phthalate Plasticizers. Environ Sci Technol 51:3902–3910. https://doi.org/10.1021/acs.est.6b05612

2. Estévez-Danta A, Rodil R, Pérez-Castaño B, Cela R, Quintana JB, González-Mariño I (2021) Comprehensive determination of phthalate, terephthalate and di-iso-nonyl cyclohexane-1,2-dicarboxylate metabolites in wastewater by solid-phase extraction and ultra(high)-performance liquid chromatography-tandem mass spectrometry. Talanta 224:. https://doi.org/10.1016/j.talanta.2020.121912

3. González-Mariño I, Ares L, Montes R, Rodil R, Cela R, López-García E, Postigo C, López de Alda M, Pocurull E, Marcé RM, Bijlsma L, Hernández F, Picó Y, Andreu V, Rico A, Valcárcel Y, Miró M, Etxebarria N, Quintana JB (2021) Assessing population exposure to phthalate plasticizers in thirteen Spanish cities through the analysis of wastewater. J Hazard Mater 401:. https://doi.org/10.1016/j.jhazmat.2020.123272

4. Kumar R, Adhikari S, Driver E, Zevitz J, Halden RU (2022) Application of wastewater-based epidemiology for estimating population-wide human exposure to phthalate esters, bisphenols, and terephthalic acid. Science of the Total Environment 847:. https://doi.org/10.1016/j.scitotenv.2022.157616

5. Tang S, He C, Thai P, Vijayasarathy S, Mackie R, Toms LML, Thompson K, Hobson P, Tscharke B, O'Brien JW, Mueller JF (2020) Concentrations of phthalate metabolites in Australian urine samples and their contribution to the per capita loads in wastewater. Environ Int 137:. https://doi.org/10.1016/j.envint.2020.105534

6. Du P, Zhou Z, Huang H, Han S, Xu Z, Bai Y, Li X (2018) Estimating population exposure to phthalate esters in major Chinese cities through wastewater-based epidemiology. Science of the Total Environment 643:1602–1609. https://doi.org/10.1016/j.scitotenv.2018.06.325

7. Alygizakis N, Galani A, Rousis NI, Aalizadeh R, Dimopoulos MA, Thomaidis NS (2021) Change in the chemical content of untreated wastewater of Athens, Greece under COVID-19 pandemic. Science of the Total Environment 799:. https://doi.org/10.1016/j.scitotenv.2021.149230

8. Senta I, Rodríguez-Mozaz S, Corominas L, Covaci A, Petrovic M (2022) Applicability of an on-line solid-phase extraction liquid chromatography – tandem mass spectrometry for the wastewater-based assessment of human exposure to chemicals from personal care and household products. Science of the Total Environment 845:. https://doi.org/10.1016/j.scitotenv.2022.157309

9. Devault DA, Karolak S, Lévi Y, Rousis NI, Zuccato E, Castiglioni S (2018) Exposure of an urban population to pesticides assessed by wastewater-based epidemiology in a Caribbean island. Science of the Total Environment 644:129–136. https://doi.org/10.1016/j.scitotenv.2018.06.250

10. Rousis NI, Zuccato E, Castiglioni S (2017) Wastewater-based epidemiology to assess human exposure to pyrethroid pesticides. Environ Int 99:213–220. https://doi.org/10.1016/j.envint.2016.11.020

11. Rousis NI, Zuccato E, Castiglioni S (2016) Monitoring population exposure to pesticides based on liquid chromatography-tandem mass spectrometry measurement of their urinary metabolites in urban wastewater: A novel biomonitoring approach. Science of the Total Environment 571:1349–1357. https://doi.org/10.1016/j.scitotenv.2016.07.036

12. Rousis NI, Gracia-Lor E, Reid MJ, Baz-Lomba JA, Ryu Y, Zuccato E, Thomas K V., Castiglioni S (2020) Assessment of human exposure to selected pesticides in Norway by wastewater analysis. Science of the Total Environment 723:. https://doi.org/10.1016/j.scitotenv.2020.138132

13. Rousis NI, Gracia-Lor E, Hernández F, Poretti F, Santos MM, Zuccato E, Castiglioni S (2021) Wastewater-based epidemiology as a novel tool to evaluate human exposure to pesticides: Triazines and organophosphates as case studies. Science of the Total Environment 793:. https://doi.org/10.1016/j.scitotenv.2021.148618

14. Rousis NI, Gracia-Lor E, Zuccato E, Bade R, Baz-Lomba JA, Castrignanò E, Causanilles A, Covaci A, de Voogt P, Hernàndez F, Kasprzyk-Hordern B, Kinyua J, McCall AK, Plósz BG, Ramin P, Ryu Y, Thomas K V., van Nuijs A,

Yang Z, Castiglioni S (2017) Wastewater-based epidemiology to assess pan-European pesticide exposure. Water Res 121:270–279. https://doi.org/10.1016/j.watres.2017.05.044

15. Been F, Bastiaensen M, Lai FY, Van Nuijs ALN, Covaci A (2017) Liquid Chromatography-Tandem Mass Spectrometry Analysis of Biomarkers of Exposure to Phosphorus Flame Retardants in Wastewater to Monitor Community-Wide Exposure. Anal Chem 89:10045–10053. https://doi.org/10.1021/acs.analchem.7b02705

16. Been F, Bastiaensen M, Lai FY, Libousi K, Thomaidis NS, Benaglia L, Esseiva P, Delémont O, Van Nuijs ALN, Covaci A (2018) Mining the Chemical Information on Urban Wastewater: Monitoring Human Exposure to Phosphorus Flame Retardants and Plasticizers. Environ Sci Technol 52:6996–7005. https://doi.org/10.1021/acs.est.8b01279

17. O'Brien JW, Thai PK, Brandsma SH, Leonards PEG, Ort C, Mueller JF (2015) Wastewater analysis of Census day samples to investigate per capita input of organophosphorus flame retardants and plasticizers into wastewater. Chemosphere 138:328–334. https://doi.org/10.1016/j.chemosphere.2015.06.014

18. Karthikraj R, Kannan K (2017) Mass loading and removal of benzotriazoles, benzothiazoles, benzophenones, and bisphenols in Indian sewage treatment plants. Chemosphere 181:216–223. https://doi.org/10.1016/j.chemosphere.2017.04.075

19. Wang H, Tang S, Zhou X, Gao R, Liu Z, Song X, Zeng F (2022) Urinary concentrations of bisphenol analogues in the south of China population and their contribution to the per capital mass loads in wastewater. Environ Res 204:. https://doi.org/10.1016/j.envres.2021.112398

20. Lopardo L, Petrie B, Proctor K, Youdan J, Barden R, Kasprzyk-Hordern B (2019) Estimation of community-wide exposure to bisphenol A via water fingerprinting. Environ Int 125:1–8. https://doi.org/10.1016/j.envint.2018.12.048

21. Xue J, Kannan K (2019) Mass flows and removal of eight bisphenol analogs, bisphenol A diglycidyl ether and its derivatives in two wastewater treatment plants in New York State, USA. Science of the Total Environment 648:442–449. https://doi.org/10.1016/j.scitotenv.2018.08.047

22. Tang S, He C, Thai PK, Heffernan A, Vijayasarathy S, Toms L, Thompson K, Hobson P, Tscharke BJ, O'Brien JW, Thomas K V., Mueller JF (2020) Urinary concentrations of bisphenols in the australian population and their association with the per capita mass loads in wastewater. Environ Sci Technol 54:10141–10148. https://doi.org/10.1021/acs.est.0c00921

23. Proctor K, Petrie B, Lopardo L, Muñoz DC, Rice J, Barden R, Arnot T, Kasprzyk-Hordern B (2021) Micropollutant fluxes in urban environment – A catchment perspective. J Hazard Mater 401:. https://doi.org/10.1016/j.jhazmat.2020.123745

24. Jeong Y, Da Silva KM, Iturrospe E, Fuiji Y, Boogaerts T, van Nuijs ALN, Koelmel J, Covaci A (2022) Occurrence and contamination profile of legacy and emerging per- and polyfluoroalkyl substances (PFAS) in Belgian wastewater using target, suspect and non-target screening approaches. J Hazard Mater 437:. https://doi.org/10.1016/j.jhazmat.2022.129378

25. Nguyen HT, McLachlan MS, Tscharke B, Thai P, Braeunig J, Kaserzon S, O'Brien JW, Mueller JF (2022) Background release and potential point sources of per- and polyfluoroalkyl substances to municipal wastewater treatment plants across Australia. Chemosphere 293:. https://doi.org/10.1016/j.chemosphere.2022.133657

26. Wang W, Kannan K (2017) Mass loading and emission of benzophenone-3 (BP-3) and its derivatives in wastewater treatment plants in New York State, USA. Science of the Total Environment 579:1316–1322. https://doi.org/10.1016/j.scitotenv.2016.11.124

27. Adhikari S, Kumar R, Driver EM, Perleberg TD, Yanez A, Johnston B, Halden RU (2022) Mass trends of parabens, triclocarban and triclosan in Arizona wastewater collected after the 2017 FDA ban on antimicrobials and during the COVID-19 pandemic. Water Res 222:. https://doi.org/10.1016/j.watres.2022.118894
